# Incidence and factors associated with unfavourable treatment outcome among patients with rifampicin-resistant pulmonary tuberculosis in Yaoundé, Cameroon

**DOI:** 10.11604/pamj.2021.38.229.28317

**Published:** 2021-03-02

**Authors:** Alain Kuaban, Adamou Dodo Balkissou, Marie Christine Essadi Ekongolo, Abdou Wouoliyou Nsounfon, Eric Walter Pefura-Yone, Christopher Kuaban

**Affiliations:** 1 Faculty of Medicine and Biomedical Sciences, The University of Yaoundé I, Yaoundé, Cameroon,; 2 Faculty of Medicine, The University of Ngaoundéré, Garoua, Cameroon,; 3 Chest Service, Jamot Hospital in Yaoundé, Yaoundé, Cameroon,; 4 Faculty of Health Sciences, The University of Bamenda, Bamenda Regional Hospital, Bamenda, Cameroon

**Keywords:** Rifampicin-resistant tuberculosis, unfavourable treatment outcome, factors associated, Yaoundé, Cameroon

## Abstract

**Introduction:**

in Cameroon patients with multidrug/rifampicin resistant pulmonary tuberculosis (MDR/RR-PTB) are treated with a 9-11 month standardised shorter treatment regimen. Despite its effectiveness, factors associated with the occurrence of an unfavourable treatment outcome in this group of patients are not known. Determine the incidence and identify factors associated with an unfavourable treatment outcome among patients with rifampicin resistant pulmonary tuberculosis (RR-PTB) in Yaoundé.

**Methods:**

we conducted a retrospective record review of all consecutive patients with bacteriologically confirmed RR-PTB followed up at the specialised MDR/RR-TB treatment centre of the Jamot Hospital in Yaoundé (JHY) from January 2013 to November 2019. A patient was classified as having an unfavourable outcome if he/she had treatment failure, died or was lost to follow-up during the course of treatment.

**Results:**

a total of 242 RR-PTB patients with a mean age of 35.59 ± 12.02 years including 144 (59.5%) males were registered. Forty-nine (49) of the 242 patients had an unfavourable treatment outcome giving a cumulative incidence of 20.20% (95% confidence interval (95% CI): 15.40-25.90%). Multivariable analysis revealed that patients with an unfavourable outcome were more likely to be males (odds ratio (OR): 2.94; 95% CI: 1.24-7.00, p= 0.015), HIV infected (OR: 2.67; 95% CI: 1.17-6.06, p = 0.019), and have a baseline haemoglobin level ≤ 10g/dl (OR: 2.87; 95% CI: 1.25-6.58, p = 0.013).

**Conclusion:**

the rate of an unfavourable treatment outcome among patients with RR-PTB at the specialised MDR/RR-TB treatment centre of the JHY is relatively high. The male sex, HIV infection and moderate to severe anaemia are independent factors associated with an unfavourable treatment outcome.

## Introduction

Tuberculosis (TB) is one of the leading infectious disease causes of death globally accounting in 2018 for an estimated 1.45 million deaths [1]. Despite the considerable efforts that have been made, its control is being hampered among other things especially in low income countries like Cameroon by difficult socioeconomic conditions, HIV infection and drug resistant TB particularly its multidrug resistant (MDR) form defined as TB resistant to at least rifampicin and isoniazid. These two drugs are the most inexpensive, the best tolerated and most effective medications for the treatment of drug susceptible TB [2]. Meanwhile it has been shown that patients harbouring rifampicin-resistant TB (RR-TB) strains have a similar bad prognosis as MDR-TB cases when treated with only first-line antituberculosis drugs [3,4]. For this reason patients with RR-TB are now treated like MDR-TB cases. The World Health Organization (WHO) treatment guidelines include the option of treating MDR/RR-TB with a standardised shorter treatment regimen (STR) of 9-12 months duration instead of an individualized regimen of at least 20 months duration in patients who have not been previously treated for more than 1 month with second-line medicines and in whom resistance to second-line injectables and fluoroquinolones has been excluded [5]. The Cameroon national tuberculosis control programme (NTCP) has been using this STR for the programmatic management of patients since 2013. The regimen with a duration of 9-11 months has an intensive phase of 4-6 months composed of the following drugs: Amikacin (Am), Moxifloxacin (Mfx), Prothionamide (Pto), Clofazimine (Cfz), Isoniazid (H), Ethambutol (E) and Pyrazinamide (Z). This is followed by a fixed duration continuation phase of 5 months with the same drugs but omitting amikacin, prothionamide and isoniazid (4-6 Am-Mfx-Pto-Cfz-H-E-Z/ 5 Mfx-Cfz-E-Z). Despite the effectiveness of this STR as shown by studies carried out in several countries including Cameroon [6,7], factors associated with the occurrence of an unfavourable outcome in this group of patients to the best of our knowledge have not been reported. The aim of this study was to determine the incidence of an unfavourable treatment outcome and to identify factors associated with it among patients with rifampicin-resistant pulmonary TB (RR-PTB) under routine care with this regimen at the specialised MDR/RR-TB treatment centre in Yaoundé, Cameroon.

## Methods

**Study setting:** the study was carried out in the Jamot Hospital in Yaoundé (JHY), the sole referral specialised MDR/RR-TB treatment facility for Yaoundé and its surrounding areas. In this facility, prior to treatment all bacteriologically confirmed MDR/RR-TB patients undergo a series of pre-treatment investigations. These consist of a clinical evaluation including anthropometric measurements (weight and height) and laboratory investigations. The laboratory investigations include a full blood count, measuring of fasting blood sugar, serum creatinine, potassium, liver enzymes, thyroid stimulating hormone (TSH) as well as HIV serology. Other investigations include a pregnancy test for women of procreative age, an electrocardiogram (ECG), audiometry and a chest X-ray. Pregnant women and patients having a baseline ECG tracing that shows a QTc space > 450ms are excluded from treatment with the regimen because they present contra-indications to some of the drugs it includes. To quantify the extent of radiographic lesions, each lung is divided into three zones and the number of zones affected in each lung is counted and recorded. During the intensive phase of treatment patients are hospitalised in the specialised MDR/RR-TB treatment centre. In this phase of treatment, serum creatinine, potassium, liver enzymes, blood sugar levels and serum TSH are monitored monthly for each patient. Also during this phase all patients perform an ECG one week after treatment start and audiometry is performed at the end of month 4 of treatment.

The continuation phase starts at month 5 after sputum smear conversion of two consecutive morning specimens of sputum examined at the end of month 4 of management. In case of no sputum smear conversion at the end of month 4, the intensive phase is continued for a maximum of 2 months with monthly evaluation of sputum smears. Treatment of patients failing to respond bacteriologically (no sputum conversion at the end of month 6 of treatment accompanied by lack of clinical improvement) is stopped and an individualized regimen when possible is used for their management. During the continuation phase of treatment, patients receive their medications daily on an ambulatory basis under the supervision of specifically dedicated health workers. Provision of social support services needed by some patients is done on a case by case basis. Clinical evaluations as well as sputum smears and cultures are performed monthly throughout the period of treatment. The outcome of patients at the end of treatment is recorded into one of the following six mutually exclusive categories according to the recommendations of The International Union Against Tuberculosis and Lung Disease (The Union) [8]: cured: treatment completed without evidence of failure and 2 consecutive cultures taken at least 30 days apart negative in the continuation phase; treatment completed: treatment completed without evidence of failure with no record that 2 consecutive cultures taken at least 30 days apart are negative in the continuation phase; died: patient who dies for any reason during the course of treatment; treatment failure: patient who has a positive culture after ≥ 6 months of treatment (except for an isolated positive culture, which is a culture preceded by ≥ 1 and followed by ≥ 2 negative cultures); lost to follow-up: a patient whose treatment was interrupted for ≥ 2 consecutive months; not evaluated: a patient for whom no treatment outcome is assigned (this includes patients “transferred out” to another treatment unit and whose treatment outcome is unknown).

**Study design and population:** the study population consisted of a retrospective cohort of all consecutive bacteriologically confirmed rifampicin resistant pulmonary tuberculosis (RR-PTB) patients aged 15 years and above that were systematically hospitalised and followed up at the specialised MDR/RR-TB treatment centre of the JHY from January 2013 to November 2019 (a total duration of 83 months). Patients lost to follow-up prior to commencement of treatment; transferred out to other specialised treatment centres as well as those with incomplete records were excluded.

**Data collection:** all consecutive RR-PTB patients treated in the specialised MDR/RR-TB treatment centre of JHY over the study period were identified through a review of the MDR/RR-TB register and patients´ treatment forms. For each patient identified, the following information at baseline was extracted from the register and treatment form and recorded on a pre-prepared data collection form: age, sex, prior TB category of patient (new, relapse, treatment failure, return after default), HIV status and CD4 cell count if patient was HIV positive, the location of TB (pulmonary with or without extrapulmonary involvement). The height in meters (m) and weight in kilograms of each patient were also extracted from his/her treatment form and the body mass index (BMI) calculated as weight (kg)/ (height in m^2^) was recorded. Equally extracted from each patient´s treatment form were the following laboratory results at baseline: serum levels of liver transaminases (AST and ALT) and haemoglobin (Hb) level. Serum liver transaminases results were considered abnormal when they were above 40IU/L. A patient was defined as having mild anaemia when the haemoglobin level was > 10g/dL and < 12 g/dL, and as having moderate and severe anaemia if the Hb levels were 7-10g/dL and < 7g/dL respectively. The results of each patient´s sputum smear examinations at baseline graded semi-quantitatively (negative, rare, 1+, 2+, 3+) as well as the sputum culture results equally at baseline and graded semi-quantitatively (contaminated, negative, rare, 1+, 2+, 3+) were also extracted and recorded. The extent of lung involvement at baseline defined by the number of lung zones affected as seen on the chest X-ray, as well as treatment outcomes (cured, treatment completed, treatment failure, death and loss follow-up) were also recorded. A patient was further classified as having an unfavourable treatment outcome if he/she had treatment failure, died during the course of treatment or was lost to follow-up.

**Data management and analysis:** data were double entered into a computer using the Epidata version 4.4.2.1 data entry software (Epidata Association, Odense, Denmark). Discordances were identified and resolved through verification of the original paper record. Data analysis was performed using the Epidata analysis software version 4.4.2.1 (Epidata Association, Odense, Denmark) and STATA (StataCorp. 2019. Stata Statistical Software: Release 16. College Station, TX: StataCorp LLC.). The cumulative incidence of an unfavourable treatment outcome was calculated as the proportion of patients with an unfavourable outcome. Chi-square or where appropriate Fisher´s exact test was used to compare proportions while the Student t-test was used to compare means. Multivariable logistic regression analysis was performed using variables found to be significantly associated or having borderline association (p-value < 0.1) with an unfavourable treatment outcome in the bivariate analysis to identify those that were independently associated with it. A p-value < 0.05 was used to characterise significant results.

**Ethical clearance:** ethical clearance for the study was obtained from the Institutional review board of the Faculty of Medicine and Biomedical Sciences of the University of Yaoundé I. Administrative authorization to carry out the study at the specialised MDR/RR-TB treatment centre of the JHY was granted by the authorities of the hospital.

## Results

**Baseline characteristics of study population:** a total of 242 records of patients with bacteriologically confirmed RR-PTB were included in the study. Of these, 144 (59.5%) were males with a mean age of 35.59 ± 12.02 years. The majority (87.6%) of the patients were previously treated patients and 80 (33.10%) of the patients were HIV infected. Extensive lung involvement by TB disease (≥ 2 lung zones) was found in 226 (94.6%) of the 237 patients for whom this was recorded. Among the 240 patients for whom information on the bacillary load was recorded, 89.2% had a bacillary load of 2+ or more. One hundred and eighty-seven (77.3%) of the 242 patients studied had information on their haemoglobin levels with a mean haemoglobin level of 10.70 ± 1.96g/dl (Table 1).

**Table 1:** baseline characteristics of the study population

Characteristic	Number (N=242)	Percentage (%)
**Gender** Male Female	144 98	59.5 40.5
Mean age group (SD) years	35.59 (12.02)	
Mean body mass index (SD), Kg/m2	19.61 (3.31)	
**Patients’ previous TB treatment** category New case Relapse after treatment with WHO category I drugs Relapse after retreatment Initial treatment failure Retreatment failure Return after default Other	30 49 21 55 56 22 9	12.4 20.2 8.7 22.7 23.1 9.1 3.7
Location of TB pulmonary and extrapulmonary	212 30	87.6 12.4
**HIV status** Negative Positive	162 80	66.9 33.1
**Extent of lung involvement on chest X-ray (N=237)** < 2 lung zones ≥ 2 lung zones	13 226	5.4 94.6
**Bacillary load at diagnosis (N= 240)** Negative Rare 1+ 2+ 3+	9 6 11 45 169	3.8 2.5 4.6 18.8 70.4
**Culture results (N= 228)** Negative or contaminated Rare 1+ 2+ 3+	6 4 25 53 140	2.6 1.8 11.0 23.2 61.4
**Audition of patients (N=237)** Normal Abnormal	97 140	40.9 59.1
Mean (SD) QTc, msec	373.83 (53.53)	
Mean (SD) ALT, IU/L	25.92 (20.11)	
Mean (SD) AST, IU/L	30.69 (23.65)	
Mean haemoglobin level, g/dl	10.70 (1.96)	

**Outcomes of treatment and incidence of an unfavourable outcome:** the distribution of the different outcomes of treatment is presented in (Table 2). Of the 242 patients, 193 (79.8%) had a favourable outcome. Forty-nine of the 242 patients studied had an unfavourable outcome (death, treatment failure, loss to follow-up) giving a cumulative incidence of an unfavourable outcome of 20.20% (95% CI=15.40-25.90).

**Table 2:** outcome of treatment of 242 pulmonary RR-TB patients studied

Treatment Outcome	Number (N=242)	Percentage (%)
Cured	181	74.8
Treatment Completed	12	5.0
Died	31	12.8
Treatment failure	10	4.1
Loss to follow up	8	3.3

**Factors associated with an unfavourable treatment outcome:** the characteristics of patients with unfavourable and favourable treatment outcomes are summarised and compared in (Table 3). Bivariate analysis revealed that an unfavourable outcome was associated with the male sex (p=0.077), HIV infection (p= 0.001), having both pulmonary and extrapulmonary TB (p= 0.053), a haemoglobin level ≤ 10g/dl (p=0.004), and abnormal levels of liver transaminases (AST, p=0.005; ALT, p=0.007). On the multivariable level including only those factors that were associated with an unfavourable outcome in bivariate analysis, only the male sex (Odds ratio (OR): 2.94; 95% Confidence interval (95% CI): 1.24-7.00, p= 0.015), HIV infection (OR: 2.67; 95% CI: 1.17-6.06, p= 0.019) and a haemoglobin level ≤ 10g/dl (OR: 2.87; 95% CI: 1.25-6.58, p= 0.013) remained significantly associated with an unfavourable treatment outcome (Table 4).

**Table 3:** comparison of baseline characteristics of pulmonary RR-TB patients with unfavourable and favourable treatment outcomes

Characteristic	Unfavourable outcome N=49 (%)	Favourable Outcome N=193 (%)	p-value
**Gender** Male Female	34 (69.4) 15 (30.0)	110 (57.0) 83 (43.0)	0.077
			
**Age group (years)** < 40 ≥ 40	21 (42.9) 28 (57.1)	68 (35.2) 125 (64.8)	0.205
**Previously treated for TB** Yes No	40 (81.6) 9 (18.4)	172 (89.1) 21 (10.9)	0.121
**Location of TB** Pulmonary and extrapulmonary Pulmonary	10 (20.4) 39 (79.6)	20 (10.4) 173 (89.6)	0.053
**HIV status** Positive Negative	26 (53.1) 23 (46.9)	54 (28.0) 139 (72.0)	0.001
Mean (SD) BMI (kg/m2)	18.96 ± 3.72	19.75 ± 3.20	0.165
**Extent of lung involvement on chest X-ray (N= 239)** ≥ 2 lung zones < 2 lung zones	44( 93.6) 3 (6.4)	182 (94.8) 10 (5.2)	0.489
**Bacillary load at diagnosis (N= 240)** ≥ 2+ < 2+	42 (87.5) 6 (12.5)	172 (89.6) 20 (10.4)	0.422
**Culture results (N= 228)** ≥ 2+ < 2+	32 (88.9) 4 (11.1)	161 (83.9) 31 (16.1)	0.314
**Audition of patients (N= 237)** Abnormal Normal	29 (64.4) 16 (35.6)	111 (57.8) 81 (42.2)	0.261
**Haemoglobin levels (g/dl) (N= 187)** ≤ 10 > 10	23 (57.8) 19 (45.2)	44 (30.3) 101 (69.7)	0.004
**AST (IU/L) (N= 241)** > 40 ≤ 40	14 (29.2) 34 (70.8)	30 (15.5) 163 (84.5)	0.029
**ALT (IU/L)(N= 241)** > 40 ≤ 40	11 (22.9) 37 (77.1)	22 (11.4) 171 (88.6)	0.038

**Table 4:** factors associated with an unfavourable treatment outcome in pulmonary RR-TB patients followed up at the specialised MDR/RR-TB treatment centre of the JHY: multivariable analysis

Factors	Odds ratio	95% Confidence interval (CI)	p-value
Male sex	2.94	1.24-7.00	0.015
HIV positivity	2.67	1.17-6.06	0.019
Both pulmonary and extrapulmonary TB	0.62	0.19-2.04	0.431
Haemoglobin level ≤ 10g/dl	2.87	1.25-6.58	0.013
AST > 40 IU/L	1.71	0.48-6.13	0.407
ALT > 40 IU/L	2.61	0.59-11.52	0.205

## Discussion

One of the most important elements in programmatic TB management is to ensure a successful antituberculosis treatment outcome according to the World Health Organization [1]. The success of antituberculosis treatment under programmatic conditions is estimated by the cure rate i.e. the proportion of patients with bacteriologically proven eradication of the bacilli. Completion of treatment until the scheduled end is also accepted as another indicator of success if no bacteriological examination can be performed at the end of treatment. A treatment success rate of at least 90% is necessary in order to lower the prevalence of the disease [1]. Altogether therefore the rate of an unfavourable treatment outcome (death, treatment failure, and loss to follow-up) should not exceed 10%. The rate of an unfavourable treatment outcome of 20.2% observed in our study is therefore far higher than the WHO recommended rate [1]. Our results are however in line with those of the study that used the 9-11 months shorter treatment regimen for the management of MDR/RR-TB in nine African countries [7]. The unfavourable treatment outcome rate observed in our study is however, much lower than the global rate of 38% reported by the WHO in 2018 [1]. The difference observed between our results and the global rate reported by the WHO could be due to the fact that the global rate of an unfavourable treatment outcome is a mix of treatment results of management with the standardised shorter treatment regimen (STR) and the long individualized treatment regimens (> 20 months). Indeed in countries where these individualized treatment regimens are used low rates of favourable outcomes ranging for example from 49% in Ukraine [1] to 71% in Pakistan [9] have been observed.

The major cause of an unfavourable treatment outcome in our study was death. Indeed out of the 49 patients with an unfavourable outcome, 31 (63.3%) died during the course of treatment (Table 3). Similar high death rates in MDR/RR-TB patients undergoing treatment have been reported by other authors [1,6,7]. In this study unfortunately, due to its retrospective design we could not be able to ascertain the specific causes of death. This notwithstanding, several reasons can be given to explain this high death rate. Firstly it may have been due to associated comorbid conditions such as advanced HIV coinfection as has been reported in several studies [7-10]. Secondly, many patients probably due to socio-economic challenges they faced might have been seeking health care when their disease was already in a far advanced stage. Indeed out of the 49 patients who had an unfavourable treatment outcome, 36 (73.5%) had 3 or more lung zones affected by the TB disease and 21 (58.3%) of these died (data not shown).

Of the factors that showed a significant or borderline relationship with an unfavourable treatment outcome in our study on bivariate analysis (Table 4), only the male sex, HIV infection and a haemoglobin level ≤ 10g/dl remained as significant independent determinants of an unfavourable outcome on multivariable analysis. Males were more likely to have an unfavourable treatment outcome than females in this study. It has been observed in several studies [11-13] that males are more likely than females to have an unfavourable treatment outcome especially due to loss to follow-up during treatment. The precise reason for this difference between males and females in loss to follow-up rates is not known. The difference between the sexes could however stem from the difficulties inherent in reconciling a salaried activity and follow-up of a lengthy course of antituberculosis treatment in men more than in women [14,15]. Secondly, according to some authors [11], males have higher rates of alcohol consumption which can lead them to adhere less to drug treatment than females. Unfortunately in our study, given that we used a secondary data source that did not have information on this variable, the role of alcohol consumption as a risk factor for an unfavourable treatment outcome could not be assessed.

In our study, it was also observed that HIV infection was an independent factor associated with an unfavourable treatment outcome. Our results are not in agreement with those of some authors as they did not observe an association between HIV infection and an unfavourable treatment outcome [6,12]. Several possible reasons can be given to explain this association found in our study. Firstly, as has been reported by other authors [16], HIV positive patients who died in our cohort of patients may have been severely immune depressed with severe extensive or disseminated disease which could lead to death. Indeed out of the 14 HIV infected people who had an unfavourable outcome in our study, 10 (71.4%) had a CD4+ cell count less than 200/mm3 (data not shown). Secondly, HIV positive patients who were lost to follow-up may have abandoned their treatment because of the heavy pill burden they had to endure for their TB/HIV coinfection. Thirdly, some of these HIV infected patients could also have abandoned treatment due to the combined use of antituberculosis drugs and antiretroviral therapy which as it is well known can lead to severe adverse reactions [17,18]. Finally, some of these patients may have had sub-therapeutic drug levels which could lead to treatment failure since it is also known that there is poor absorption of antituberculosis drugs in HIV infected patients [19].

In this study also an unfavourable outcome was equally found to be common in patients who had moderate to severe anaemia. Our result is in line with those reported by several authors [17,20-22]. The precise reason why anaemia should be associated to an unfavourable outcome is still unclear. It has, however, been suggested that anaemia could contribute to the progression of TB disease, as well as the occurrence of treatment failure and death [17]. Also, severe anaemia coupled with extensive TB lung disease can lead to cardiorespiratory failure and death. This not withstanding, some authors have stated that the independent nature of the effect of anaemia cannot be ascertained as in their series the majority of their patients were equally malnourished [17,20]. In our study however, malnutrition did not appear to be associated with unfavourable outcome. Our study was limited by its retrospective design and the operational nature of the study relying on records routinely maintained in the specialised MDR/RR-TB treatment centre of the JHY. However, we think that the supervision and monitoring of this centre by the national tuberculosis control programme is robust. As such the retrospective nature of our study is likely to have only a minimal impact on our results. Secondly as this study was only conducted in one of the specialised treatment centres for MDR/RR-TB, our results may not be easily generalised to all the MDR/RR-TB patients in Cameroon. This not withstanding, the strength of our study lies in the fact that it is the first study to assess the occurrence of an unfavourable treatment outcome and factors associated with it in patients with bacteriologically confirmed RR-TB in a major specialised MDR/RR-TB treatment centre in Cameroon.

## Conclusion

The rate of an unfavourable treatment outcome among patients with bacteriologically confirmed RR-PTB, followed-up in the specialised MDR/RR-TB treatment centre of the JHY is relatively high. The male sex, HIV infection and moderate to severe anaemia are the most important predictors of an unfavourable treatment outcome in our cohort. As such RR-PTB patients with these conditions initiating treatment at baseline should be closely managed and supervised by health care givers in a bid to reduce the occurrence of an unfavourable outcome.

### What is known about this topic

- The effectiveness of the shorter standardised treatment regimen for the management of patients with bacteriologically confirmed pulmonary rifampicin resistant tuberculosis is known;
- To the best of our knowledge no evidence exists on the factors that can be associated with the occurrence of unfavourable outcome among patients with rifampicin-resistant pulmonary TB (RR-PTB) under routine care with the shorter standardised treatment regimen.

### What this study adds

- The rate of an unfavourable treatment outcome among patients with bacteriologically confirmed rifampicin resistant pulmonary TB, followed-up in a major specialised MDR/RR-TB treatment centre in Cameroon is relatively high;
- The male sex, HIV infection and moderate to severe anaemia are the most important predictors of an unfavourable treatment outcome in these patients.
